# A new Mo^VI^ Schiff base complex: methanol[*N*′-(3-meth­oxy-2-oxidobenzyl­idene)benzohydrazidato]dioxido­molybdenum(VI)

**DOI:** 10.1107/S1600536811020101

**Published:** 2011-06-04

**Authors:** Iran Sheikhshoaie, Vratislav Langer, Seyed Ali Yasrebi

**Affiliations:** a Department of Chemistry, Shahid Bahonar University, Kerman, Iran; b Environmental Inorganic Chemistry, Department of Chemical and Biological Engineering, Chalmers University of Technology, SE-41296 Göteborg, Sweden

## Abstract

In the title benzil­idene Schiff base molybdenum(VI) complex, [Mo(C_15_H_12_N_2_O_3_)O_2_(CH_3_OH)], the Mo^VI^ ion is coordinated by two oxide O atoms and by two O atoms and one N atom of the tridentate *N*′-(3-meth­oxy-2-oxidobenzyl­idene)benzo­hydrazidate (*L*) Schiff base ligand. The methanol O atom completes the distorted octa­hedral configuration of the Mo^VI^ atom. Strong O—H⋯N hydrogen bonds form a *C*(5) chain around a 2_1_ screw axis. Weak C—H—O hydrogen bonds are also present.

## Related literature

For general background, see: Alizadeh *et al.* (1999 ▶); Ambroziak *et al.* (2004 ▶); Archer & Wang (1990 ▶); Bagherzadeh & Amini (2009 ▶); Bagherzadeh *et al.* (2008 ▶); Bhatia *et al.* (1981 ▶); Bindlish *et al.* (1978 ▶); Blake *et al.* (1995 ▶); Chang *et al.* (1998 ▶); Costamagna *et al.* (1992 ▶); Dhar & Taploo (1982 ▶); Hatefi *et al.* (2009 ▶); Holm (1990 ▶); Jalali-Heravi *et al.* (1999 ▶); Johnson *et al.* (1996 ▶); Maurya *et al.* (1997 ▶); Sheikhshoaie & Fabian (2009 ▶); Yamada (1999 ▶). For details of the synthesis, see: Perrin *et al.* (1990 ▶). For related structures, see: Dinda *et al.* (2006 ▶); Glowiak *et al.* (2003 ▶); Liimatainen *et al.* (2000 ▶); Monadi *et al.* (2009 ▶); Niaz *et al.* (2010 ▶); Pramaniky *et al.* (2007 ▶); Rao *et al.* (1999 ▶); Rezaeifard *et al.* (2010 ▶); Saeednia *et al.* (2009 ▶); Sheikhshoaie *et al.* (2009 ▶); Vrdoljak *et al.* (2010 ▶).
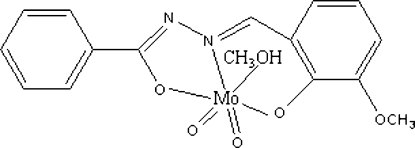


## Experimental

### 

#### Crystal data

[Mo(C_15_H_12_N_2_O_3_)O_2_(CH_4_O)]
*M* *_r_* = 428.25
Monoclinic, 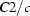

*a* = 29.400 (13) Å
*b* = 8.553 (4) Å
*c* = 14.391 (6) Å
β = 112.993 (8)°
*V* = 3331 (2) Å^3^
*Z* = 8
Mo *K*α radiation
μ = 0.82 mm^−1^
*T* = 173 K
0.58 × 0.54 × 0.46 mm

#### Data collection

Bruker SMART CCD diffractometer
Absorption correction: multi-scan (*SADABS*; Sheldrick, 2003 ▶) *T*_min_ = 0.368, *T*_max_ = 0.703
27788 measured reflections
5867 independent reflections
4401 reflections with *I* > 2σ(*I*)
*R*_int_ = 0.065

#### Refinement

*R*[*F* ^2^ > 2σ(*F* ^2^)] = 0.038
*wR*(*F* ^2^) = 0.104
*S* = 1.01
5867 reflections
229 parameters
H-atom parameters constrained
Δρ_max_ = 1.38 e Å^−3^
Δρ_min_ = −1.80 e Å^−3^

### 

Data collection: *SMART* (Bruker, 2003 ▶); cell refinement: *SAINT* (Bruker, 2003 ▶); data reduction: *SAINT* and *SADABS* (Sheldrick, 2003 ▶); program(s) used to solve structure: *SHELXS97* (Sheldrick, 2008 ▶); program(s) used to refine structure: *SHELXL97* (Sheldrick, 2008 ▶); molecular graphics: *DIAMOND* (Brandenburg, 2010 ▶); software used to prepare material for publication: *SHELXTL* (Sheldrick, 2008 ▶) and *PLATON* (Spek, 2009 ▶).

## Supplementary Material

Crystal structure: contains datablock(s) I, global. DOI: 10.1107/S1600536811020101/dn2692sup1.cif

Structure factors: contains datablock(s) I. DOI: 10.1107/S1600536811020101/dn2692Isup2.hkl

Additional supplementary materials:  crystallographic information; 3D view; checkCIF report

## Figures and Tables

**Table 1:** Selected geometric parameters (Å, °)

Mo1—O1	2.0281 (19)
Mo1—O2	1.9391 (17)
Mo1—O4	1.7096 (18)
Mo1—O5	1.7093 (19)
Mo1—N1	2.248 (2)
Mo1—O1*M*	2.3374 (18)

**Table 2:** Hydrogen-bond geometry (Å, °)

*D*—H⋯*A*	*D*—H	H⋯*A*	*D*⋯*A*	*D*—H⋯*A*
O1*M*—H1*M*⋯N2^i^	0.84	1.87	2.700 (3)	173
C1*M*—H1*BM*⋯O1^ii^	0.98	2.58	3.402 (3)	141
C6—H6⋯O4^iii^	0.95	2.53	3.450 (3)	162
C11—H11⋯O1	0.95	2.44	2.767 (3)	100
C15—H15⋯O1*M*^ii^	0.95	2.54	3.427 (3)	156

Symmetry codes: (i) 
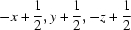
; (ii) 
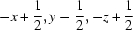
; (iii) 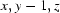.

**Fig. 1.**
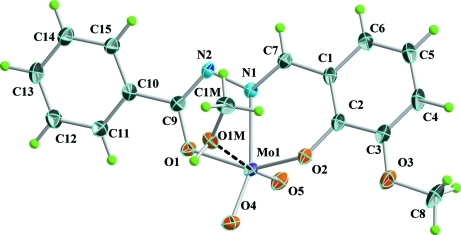
The atom numbering scheme for (I), with atomic displacement ellipsoids drawn at the 50% probability level.

**Fig. 2.**
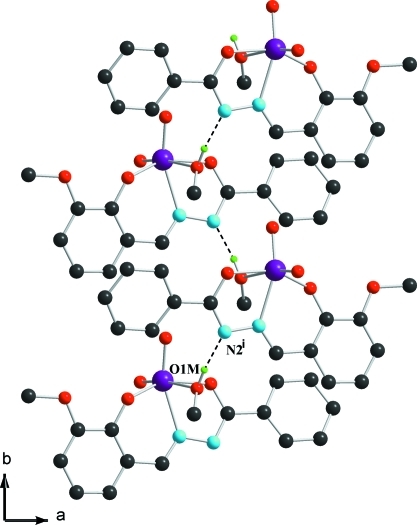
The hydrogen-pattern (dashed lines) in (I). C(5) chains are formed in the b-direction. Symmetry code: (i):-x + 1/2,y + 1/2,-z + 1/2.

**Fig. 3.**
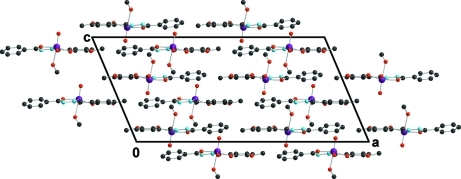
Content of the unit cell for (I) in projection along the b axis.

## supplementary crystallographic information

### Comment

The condensation products of primary amines and aldehydes or ketones (RCH=NR'), where *R* and *R*' represent alkyl and /or aryl constituents), are called Schiff bases (Dhar & Taploo, 1982); they play an important role in inorganic chemistry (Blake *et al.*, 1995; Johnson *et al.*,1996; Alizadeh *et al.*, 1999) as they easily form stable complexes with most transition metal ions. Transition metal compounds containing the Schiff base ligands have been of interest for many years (Yamada, 1999; Chang *et al.*, 1998; Archer & Wang, 1990; Sheikhshoaie & Fabian, 2009; Jalali-Heravi *et al.*, 1999). Many complexes play an important role in the developing of coordination chemistry related to catalytic (Holm, 1990; Rao *et al.*, 1999; Bagherzadeh *et al.*, 2008; Ambroziak *et al.*, 2004; Bagherzadeh & Amini, 2009; Hatefi *et al.*, 2009) and enzymatic reactions (Bindlish *et al.*, 1978; Bhatia *et al.*, 1981; Costamagna *et al.*, 1992). High valent metal oxo species have demonstrated the ability to catalyze the oxidation of a variety of organic substrates, *via* homogeneous as well as heterogeneous routes (Holm, 1990; Maurya *et al.*, 1997). In particular, the Mo-catalyzed alkene to epoxide conversion has received most attention. In the course of our studies on transition metal Schiff base complexes (Sheikhshoaie *et al.*, 2009; Monadi *et al.*, 2009), we have synthesized a dioxo molybdenium complex with a tridentate ligand (*L*) and the crystal structure of the title complex (I) is presented here.

The molecular structure of (I) is shown in Fig. 1. and the selected bond distances and angles are given in Table 1. The calculated bond valence by *PLATON* (version 91110: Spek, 2009) for Mo atom is 5.89, *i.e.* it exhibits the oxidation state +VI. Mo atom is surrounded by two O atoms and one N atom of the tridentate Schiff base ligand 3-methoxysalicylidenbenzoyl hydrazine in a distorted octahedral configuration. The Mo—O distances of the oxo ligands (O4 and O5) are significantly shorter [average 1.7094 (18) Å] than the corresponding distances to the O atoms of the tridentate ligands (O1 and O2) [average 1.98 (6) Å]. The Mo—N distance is longer [2.248 (2) Å] and the distance to methanol O atom is longest.

The aromatic ring C1/C2/C3/C4/C5/C6 is slightly distorted (χ^2^ = 56.6), while the phenyl ring C10/C11/C12/C13/C14/C15 is planar (χ^2^ = 4.7); the dihedral angle between them is 23.09 (12)*^o^*.

There are strong hydrogen bonds present between the hydroxy group of methanol and N2^i^ atom in an adjacent complex [symmetry code: (i):-*x* + 1/2,*y* + 1/2,-*z* + 1/2] forming thus a C(5) chain around 2_1_ screw axis, see Fig.2. There are also weak hydrogen bonds of the type in the C—H—O hydrogen bonds present, see Table 2. Fig. 3 shows the packing of the complexes in the unit cell, but there are no π-π interactions present.

Comparison with other similar structures is presented in Table 3. In all complexes molybdenum atom is coordinated by two oxo oxygen atoms, one nitrogen atom of C=N group and one oxygen atom from solvent (methanol or ethanol). In all these complexes, the longest bond from the solvent coordination indicates the most labile site available for substitution. In addition, in all the investigated complexes the bonds between molybdenium atom and nitrogen atom of imine group (C=N) are long (average: 2.26 (2) Å). Two oxo oxygen atoms are almost in *cis* position towards each other in all the cases [the average of the O=Mo=O angles: 106.1 (5)°]. The angles of N—Mo=O in the complexes indicate that one of the oxo oxygen atoms is in *cis* and the other one is in *trans* position toward nitrogen atom of imine group. The coordination geometry around Mo atoms is highly distorted octahedral in [MoO_2_(*L*) (solvent)] complexes, where (*L*) is a tridentate ligand containing an imine group and MeOH or EtOH as a solvent. Table 4 provides a comparison of important frequencies in the IR spectra of (I) and some dioxo-molybdenum(VI) complexes.

### Experimental

All reagents were used assupplied by Fluka and Merck without further purification. Solvents used for the reaction were purified and dried by conventional methods (Perrin *et al.*, 1990).

NMR spectra were obtained on a BRUKER AVANCE DRX500 (500 MHz) spectrometer. Proton chemical shifts δ are reported in p.p.m. relative to an internal standard of Me_4_Si. Elemental analyses were performed by using Heraeus CHN-O-RAPID elemental analyzer. IR spectra were recorded in KBr pellets using Shimadzu 435 spectrophotometer.

Synthesis of ligand (*L*): To a solution of 0.136 g(0.001 mol) benzohydrazide in 20 ml e thanol was added a solution of 0.152 g(0.001 mol) 3-methoxysalicylaldehyde in 10 ml e thanol, and refluxed for 5 h. The solvent was evaporated on a rotatory evaporator and the solid dissolved in 10 ml me thanol and filtered off. The filtrate was left overnight to give yellow crystals. Yield: 202 mg (75%). *Anal*. Calc. for C_15_H_14_N_2_O_3_: C, 66.66; H, 5.18; N, 10.37. Found: C, 66.79; H, 5.20; N, 10.39%. IR (KBr, cm^-1^) ν_max_ 1652.9 (s, C=O), 1614 (m, C=N). ^1^H NMR: (d_6_-DMSO): δ 3.81 (s, 3H, methoxy group), δ 6.84–7.96 (m, 8H, ArH), δ 8.671 (s, 1H, –CH=N– group), δ 11.02 (s, 1H, OH group), δ 12.09 (s, 1H, NH group).

Synthesis of the title metal complex (I) [Mo O_2_(*L*)(CH_3_OH)]: The title dioxomolybdenum(VI) complex was prepared by mixing MoO_2_(acac)_2_ (acac=.acetylacetonate) with the ligand in a 1:1 molar ratio using 30 ml dry methanol as a solvent, followed by refluxing the solution for 4 h. Deeply orange crystals were collected by filtration and dried in the room temperature, yield: 83%. ^1^H NMR: δ 3.80 (s, 3H, metoxy group), δ 7.006–8.01 (m, 8 H, Ar H), δ 8.92 (s, 1H, –CH=N– group), δ 4.08–4.11 (s, 1H, OH methanol group); δ 3.16 (d, 3H CH_3_ in methanol). IR spectra are presented in Table 4 [frequencies ν(N—H) and ν(C=O) were not observed].

### Refinement

Aromatic hydrogen atoms were refined isotropically with *U*_iso_(H) = 1.2*U*_eq_(C), and their positions were constrained to an ideal geometry using an appropriate riding model, (C—H = 0.95 Å). For methyl groups, O—C—H angles (109.5°) were kept fixed, while the torsion angle was allowed to refine with the starting positions based on the circular Fourier synthesis averaged using the local 3-fold axis with *U*_iso_(H)= 1.5*U*_eq_(C) and a constrained C—H distance of 0.98Å was applied. For hydroxy group, the O—H distance (0.84 Å) and C—O—H angle (109.5°) were kept fixed, while the torsion angle was allowed to refine with the starting position based on the maximum on the circular Fourier synthesis, *U*_iso_(H)= 1.5*U*_eq_(O).

### Crystal data

**Table:**

[Mo(C_15_H_12_N_2_O_3_)O_2_(CH_4_O)]	*F*(000) = 1728
*M**_r_* = 428.25	*D*_x_ = 1.708 Mg m^−^^3^
Monoclinic, *C*2/*c*	Mo *K*α radiation, λ = 0.71073 Å
Hall symbol: -C 2yc	Cell parameters from 5066 reflections
*a* = 29.400 (13) Å	θ = 2.5–32.3°
*b* = 8.553 (4) Å	µ = 0.82 mm^−^^1^
*c* = 14.391 (6) Å	*T* = 173 K
β = 112.993 (8)°	Block, orange
*V* = 3331 (2) Å^3^	0.58 × 0.54 × 0.46 mm
*Z* = 8	

### Data collection

**Table:**

Bruker SMART CCD diffractometer	5867 independent reflections
Radiation source: fine-focus sealed tube	4401 reflections with *I* > 2σ(*I*)
graphite	*R*_int_ = 0.065
ω scans	θ_max_ = 32.8°, θ_min_ = 2.5°
Absorption correction: multi-scan (*SADABS*; Sheldrick, 2003)	*h* = −43→43
*T*_min_ = 0.368, *T*_max_ = 0.703	*k* = −12→12
27788 measured reflections	*l* = −21→21

### Refinement

**Table:**

Refinement on *F*^2^	Primary atom site location: structure-invariant direct methods
Least-squares matrix: full	Secondary atom site location: difference Fourier map
*R*[*F*^2^ > 2σ(*F*^2^)] = 0.038	Hydrogen site location: inferred from neighbouring sites
*wR*(*F*^2^) = 0.104	H-atom parameters constrained
*S* = 1.01	*w* = 1/[σ^2^(*F*_o_^2^) + (0.0566*P*)^2^ + 2.343*P*] where *P* = (*F*_o_^2^ + 2*F*_c_^2^)/3
5867 reflections	(Δ/σ)_max_ = 0.001
229 parameters	Δρ_max_ = 1.38 e Å^−^^3^
0 restraints	Δρ_min_ = −1.80 e Å^−^^3^

### Special details

**Table:**

Experimental. Data were collected at 173 K using a Siemens *SMART* CCD diffractometer equipped with LT-2 A cooling device. A full sphere of reciprocal space was scanned by 0.3° steps in ω with a crystal–to–detector distance of 3.97 cm, 1 second per frame. Preliminary orientation matrix was obtained from the first 100 frames using *SMART* (Bruker, 2003). The collected frames were integrated using the preliminary orientation matrix which was updated every 100 frames. Final cell parameters were obtained by refinement on the position of 5066 reflections with I>10σ(I) after integration of all the frames data using *SAINT* (Bruker, 2003).
Geometry. All e.s.d.'s (except the e.s.d. in the dihedral angle between two l.s. planes) are estimated using the full covariance matrix. The cell e.s.d.'s are taken into account individually in the estimation of e.s.d.'s in distances, angles and torsion angles; correlations between e.s.d.'s in cell parameters are only used when they are defined by crystal symmetry. An approximate (isotropic) treatment of cell e.s.d.'s is used for estimating e.s.d.'s involving l.s. planes.
Refinement. Refinement of *F*^2^ against ALL reflections. The weighted *R*-factor *wR* and goodness of fit *S* are based on *F*^2^, conventional *R*-factors *R* are based on *F*, with *F* set to zero for negative *F*^2^. The threshold expression of *F*^2^ > σ(*F*^2^) is used only for calculating *R*-factors(gt) *etc*. and is not relevant to the choice of reflections for refinement. *R*-factors based on *F*^2^ are statistically about twice as large as those based on *F*, and *R*- factors based on ALL data will be even larger.

### Fractional atomic coordinates and isotropic or equivalent isotropic displacement parameters (Å^2^)

**Table:**

	*x*	*y*	*z*	*U*_iso_*/*U*_eq_	
Mo1	0.169567 (7)	0.55438 (2)	0.093970 (13)	0.01640 (7)	
O1	0.23804 (6)	0.54087 (18)	0.09191 (12)	0.0191 (3)	
O2	0.11949 (6)	0.47242 (19)	0.13716 (13)	0.0209 (3)	
O3	0.02944 (7)	0.4772 (2)	0.13247 (15)	0.0299 (4)	
O4	0.17354 (6)	0.74754 (19)	0.12674 (13)	0.0244 (4)	
O5	0.13736 (7)	0.5485 (2)	−0.03385 (13)	0.0254 (4)	
N1	0.19324 (7)	0.3025 (2)	0.11307 (13)	0.0163 (3)	
N2	0.24201 (7)	0.2753 (2)	0.12187 (14)	0.0176 (4)	
C1	0.11885 (8)	0.1902 (3)	0.12040 (16)	0.0185 (4)	
C2	0.09645 (8)	0.3331 (3)	0.12693 (16)	0.0187 (4)	
C3	0.04804 (9)	0.3332 (3)	0.12641 (17)	0.0226 (4)	
C4	0.02337 (9)	0.1919 (3)	0.11911 (19)	0.0285 (5)	
H4	−0.0093	0.1918	0.1170	0.034*	
C5	0.04618 (10)	0.0501 (3)	0.1148 (2)	0.0282 (5)	
H5	0.0291	−0.0456	0.1111	0.034*	
C6	0.09347 (9)	0.0485 (3)	0.11585 (19)	0.0236 (5)	
H6	0.1089	−0.0482	0.1135	0.028*	
C7	0.16837 (8)	0.1801 (3)	0.12059 (16)	0.0189 (4)	
H7	0.1832	0.0799	0.1264	0.023*	
C8	−0.02257 (10)	0.4859 (4)	0.1098 (2)	0.0357 (6)	
H8A	−0.0409	0.4339	0.0452	0.054*	
H8B	−0.0327	0.5958	0.1053	0.054*	
H8C	−0.0297	0.4340	0.1633	0.054*	
C9	0.26226 (8)	0.4068 (3)	0.11017 (16)	0.0171 (4)	
C10	0.31418 (8)	0.4117 (3)	0.11898 (17)	0.0186 (4)	
C11	0.33256 (9)	0.5503 (3)	0.09429 (18)	0.0212 (4)	
H11	0.3115	0.6383	0.0706	0.025*	
C12	0.38163 (10)	0.5592 (3)	0.1044 (2)	0.0265 (5)	
H12	0.3938	0.6532	0.0874	0.032*	
C13	0.41308 (10)	0.4306 (3)	0.1396 (2)	0.0279 (5)	
H13	0.4466	0.4370	0.1470	0.033*	
C14	0.39471 (9)	0.2927 (3)	0.1637 (2)	0.0277 (5)	
H14	0.4158	0.2048	0.1873	0.033*	
C15	0.34575 (9)	0.2829 (3)	0.15337 (18)	0.0228 (5)	
H15	0.3336	0.1883	0.1697	0.027*	
O1M	0.21855 (6)	0.51974 (19)	0.26524 (12)	0.0200 (3)	
H1M	0.2288	0.6039	0.2964	0.030*	
C1M	0.21418 (10)	0.4038 (3)	0.33394 (17)	0.0240 (5)	
H1AM	0.2338	0.4359	0.4035	0.036*	
H1BM	0.2263	0.3030	0.3206	0.036*	
H1CM	0.1794	0.3935	0.3244	0.036*	

### Atomic displacement parameters (Å^2^)

**Table:**

	*U*^11^	*U*^22^	*U*^33^	*U*^12^	*U*^13^	*U*^23^
Mo1	0.01570 (10)	0.01803 (10)	0.01522 (9)	0.00265 (7)	0.00577 (7)	0.00205 (7)
O1	0.0194 (8)	0.0192 (8)	0.0205 (7)	0.0034 (6)	0.0098 (6)	0.0041 (6)
O2	0.0171 (8)	0.0222 (8)	0.0249 (8)	0.0010 (6)	0.0099 (7)	−0.0004 (6)
O3	0.0191 (9)	0.0358 (10)	0.0363 (10)	0.0057 (7)	0.0126 (8)	0.0020 (8)
O4	0.0271 (9)	0.0205 (8)	0.0273 (9)	0.0058 (7)	0.0126 (7)	0.0045 (7)
O5	0.0224 (9)	0.0332 (10)	0.0186 (8)	0.0054 (7)	0.0058 (7)	0.0033 (7)
N1	0.0156 (9)	0.0190 (8)	0.0143 (8)	0.0003 (7)	0.0058 (7)	−0.0004 (6)
N2	0.0152 (8)	0.0213 (9)	0.0167 (8)	0.0019 (7)	0.0066 (7)	−0.0006 (7)
C1	0.0159 (10)	0.0238 (11)	0.0145 (9)	−0.0014 (8)	0.0046 (8)	−0.0003 (8)
C2	0.0159 (10)	0.0254 (11)	0.0138 (9)	0.0005 (8)	0.0047 (8)	0.0019 (8)
C3	0.0163 (10)	0.0308 (12)	0.0197 (10)	0.0008 (9)	0.0060 (8)	0.0007 (9)
C4	0.0172 (11)	0.0400 (14)	0.0291 (12)	−0.0050 (10)	0.0099 (10)	0.0022 (11)
C5	0.0220 (12)	0.0321 (13)	0.0308 (13)	−0.0080 (10)	0.0107 (10)	−0.0001 (10)
C6	0.0212 (11)	0.0248 (11)	0.0234 (11)	−0.0041 (9)	0.0074 (9)	−0.0007 (9)
C7	0.0177 (10)	0.0217 (10)	0.0163 (9)	0.0008 (8)	0.0055 (8)	0.0000 (8)
C8	0.0194 (12)	0.0552 (18)	0.0344 (14)	0.0113 (12)	0.0125 (11)	0.0076 (13)
C9	0.0184 (10)	0.0207 (10)	0.0115 (8)	0.0011 (8)	0.0050 (8)	−0.0002 (7)
C10	0.0181 (10)	0.0236 (11)	0.0154 (9)	−0.0003 (8)	0.0079 (8)	−0.0022 (8)
C11	0.0208 (11)	0.0233 (11)	0.0209 (10)	0.0015 (9)	0.0095 (9)	0.0031 (9)
C12	0.0248 (12)	0.0288 (12)	0.0285 (12)	−0.0037 (10)	0.0133 (10)	0.0024 (10)
C13	0.0198 (11)	0.0356 (14)	0.0304 (13)	−0.0017 (10)	0.0120 (10)	−0.0005 (10)
C14	0.0214 (12)	0.0304 (13)	0.0335 (13)	0.0034 (10)	0.0130 (10)	0.0012 (10)
C15	0.0205 (11)	0.0228 (11)	0.0280 (12)	0.0021 (9)	0.0128 (9)	0.0023 (9)
O1M	0.0241 (8)	0.0205 (8)	0.0152 (7)	−0.0036 (6)	0.0076 (6)	−0.0017 (6)
C1M	0.0307 (13)	0.0247 (11)	0.0149 (10)	0.0001 (9)	0.0071 (9)	0.0027 (8)

### Geometric parameters (Å, °)

**Table:**

Mo1—O1	2.0281 (19)	C6—H6	0.9500
Mo1—O2	1.9391 (17)	C7—H7	0.9500
Mo1—O4	1.7096 (18)	C8—H8A	0.9800
Mo1—O5	1.7093 (19)	C8—H8B	0.9800
Mo1—N1	2.248 (2)	C8—H8C	0.9800
Mo1—O1M	2.3374 (18)	C9—C10	1.482 (3)
O1—C9	1.321 (3)	C10—C15	1.400 (3)
O2—C2	1.350 (3)	C10—C11	1.404 (3)
O3—C3	1.364 (3)	C11—C12	1.394 (4)
O3—C8	1.435 (3)	C11—H11	0.9500
N1—C7	1.306 (3)	C12—C13	1.399 (4)
N1—N2	1.409 (3)	C12—H12	0.9500
N2—C9	1.313 (3)	C13—C14	1.396 (4)
C1—C2	1.409 (3)	C13—H13	0.9500
C1—C6	1.411 (3)	C14—C15	1.391 (3)
C1—C7	1.457 (3)	C14—H14	0.9500
C2—C3	1.420 (3)	C15—H15	0.9500
C3—C4	1.392 (4)	O1M—C1M	1.441 (3)
C4—C5	1.399 (4)	O1M—H1M	0.8400
C4—H4	0.9500	C1M—H1AM	0.9800
C5—C6	1.384 (4)	C1M—H1BM	0.9800
C5—H5	0.9500	C1M—H1CM	0.9800
			
O4—Mo1—O5	105.93 (8)	N1—C7—H7	118.5
O4—Mo1—O2	103.86 (8)	C1—C7—H7	118.5
O5—Mo1—O2	99.42 (9)	O3—C8—H8A	109.5
O4—Mo1—O1	95.61 (7)	O3—C8—H8B	109.5
O5—Mo1—O1	96.80 (8)	H8A—C8—H8B	109.5
O2—Mo1—O1	150.03 (7)	O3—C8—H8C	109.5
O4—Mo1—N1	155.24 (8)	H8A—C8—H8C	109.5
O5—Mo1—N1	96.81 (7)	H8B—C8—H8C	109.5
O2—Mo1—N1	81.49 (7)	N2—C9—O1	122.2 (2)
O1—Mo1—N1	71.67 (6)	N2—C9—C10	121.1 (2)
C9—O1—Mo1	120.49 (14)	O1—C9—C10	116.7 (2)
C2—O2—Mo1	134.07 (14)	C15—C10—C11	119.1 (2)
C3—O3—C8	116.8 (2)	C15—C10—C9	121.7 (2)
C7—N1—N2	116.36 (18)	C11—C10—C9	119.2 (2)
C7—N1—Mo1	128.46 (15)	C12—C11—C10	120.3 (2)
N2—N1—Mo1	115.13 (13)	C12—C11—H11	119.9
C9—N2—N1	110.11 (18)	C10—C11—H11	119.9
C2—C1—C6	119.7 (2)	C11—C12—C13	120.4 (2)
C2—C1—C7	122.9 (2)	C11—C12—H12	119.8
C6—C1—C7	117.3 (2)	C13—C12—H12	119.8
O2—C2—C1	123.0 (2)	C14—C13—C12	119.3 (2)
O2—C2—C3	117.3 (2)	C14—C13—H13	120.4
C1—C2—C3	119.6 (2)	C12—C13—H13	120.4
O3—C3—C4	125.4 (2)	C15—C14—C13	120.6 (2)
O3—C3—C2	115.2 (2)	C15—C14—H14	119.7
C4—C3—C2	119.4 (2)	C13—C14—H14	119.7
C3—C4—C5	120.8 (2)	C14—C15—C10	120.4 (2)
C3—C4—H4	119.6	C14—C15—H15	119.8
C5—C4—H4	119.6	C10—C15—H15	119.8
C6—C5—C4	120.3 (2)	C1M—O1M—H1M	109.5
C6—C5—H5	119.9	O1M—C1M—H1AM	109.5
C4—C5—H5	119.9	O1M—C1M—H1BM	109.5
C5—C6—C1	120.2 (2)	H1AM—C1M—H1BM	109.5
C5—C6—H6	119.9	O1M—C1M—H1CM	109.5
C1—C6—H6	119.9	H1AM—C1M—H1CM	109.5
N1—C7—C1	123.0 (2)	H1BM—C1M—H1CM	109.5
			
O4—Mo1—O1—C9	152.56 (16)	O2—C2—C3—C4	−178.1 (2)
O5—Mo1—O1—C9	−100.61 (16)	C1—C2—C3—C4	−0.2 (3)
O2—Mo1—O1—C9	21.8 (2)	O3—C3—C4—C5	−179.1 (2)
N1—Mo1—O1—C9	−5.67 (15)	C2—C3—C4—C5	1.6 (4)
O4—Mo1—O2—C2	174.04 (19)	C3—C4—C5—C6	−1.2 (4)
O5—Mo1—O2—C2	64.9 (2)	C4—C5—C6—C1	−0.6 (4)
O1—Mo1—O2—C2	−56.9 (3)	C2—C1—C6—C5	1.9 (3)
N1—Mo1—O2—C2	−30.6 (2)	C7—C1—C6—C5	−179.6 (2)
O4—Mo1—N1—C7	120.7 (2)	N2—N1—C7—C1	175.46 (18)
O5—Mo1—N1—C7	−82.56 (19)	Mo1—N1—C7—C1	−1.8 (3)
O2—Mo1—N1—C7	15.99 (19)	C2—C1—C7—N1	−9.5 (3)
O1—Mo1—N1—C7	−177.5 (2)	C6—C1—C7—N1	172.0 (2)
O4—Mo1—N1—N2	−56.6 (2)	N1—N2—C9—O1	−0.6 (3)
O5—Mo1—N1—N2	100.14 (14)	N1—N2—C9—C10	178.14 (18)
O2—Mo1—N1—N2	−161.31 (14)	Mo1—O1—C9—N2	5.8 (3)
O1—Mo1—N1—N2	5.20 (13)	Mo1—O1—C9—C10	−173.01 (14)
C7—N1—N2—C9	178.27 (19)	N2—C9—C10—C15	−8.8 (3)
Mo1—N1—N2—C9	−4.1 (2)	O1—C9—C10—C15	170.0 (2)
Mo1—O2—C2—C1	30.2 (3)	N2—C9—C10—C11	172.5 (2)
Mo1—O2—C2—C3	−151.97 (17)	O1—C9—C10—C11	−8.6 (3)
C6—C1—C2—O2	176.2 (2)	C15—C10—C11—C12	−0.3 (3)
C7—C1—C2—O2	−2.2 (3)	C9—C10—C11—C12	178.3 (2)
C6—C1—C2—C3	−1.5 (3)	C10—C11—C12—C13	−0.2 (4)
C7—C1—C2—C3	−179.9 (2)	C11—C12—C13—C14	0.5 (4)
C8—O3—C3—C4	−12.9 (4)	C12—C13—C14—C15	−0.3 (4)
C8—O3—C3—C2	166.5 (2)	C13—C14—C15—C10	−0.3 (4)
O2—C2—C3—O3	2.5 (3)	C11—C10—C15—C14	0.5 (3)
C1—C2—C3—O3	−179.6 (2)	C9—C10—C15—C14	−178.1 (2)

### Hydrogen-bond geometry (Å, °)

**Table:**

*D*—H···*A*	*D*—H	H···*A*	*D*···*A*	*D*—H···*A*
O1M—H1M···N2^i^	0.84	1.87	2.700 (3)	173
C1M—H1BM···O1^ii^	0.98	2.58	3.402 (3)	141
C6—H6···O4^iii^	0.95	2.53	3.450 (3)	162
C11—H11···O1	0.95	2.44	2.767 (3)	100
C15—H15···O1M^ii^	0.95	2.54	3.427 (3)	156

Symmetry codes: (i) −*x*+1/2, *y*+1/2, −*z*+1/2; (ii) −*x*+1/2, *y*−1/2, −*z*+1/2; (iii) *x*, *y*−1, *z*.

### Table 3 Comparison of Mo═O and Mo—N bond lengths (Å), N—Mo═O and O═Mo═O (°) and Mo—O (solvent)^1^ interactions (Å) in some related dioxidomolybdenium(VI) complexes

**Table:**

Cis-dioxo molybdenum complex		Mo=O	Mo-N	Mo–O(solv)^1^	N—Mo=O	O=Mo=O
[MoO_2_(bnms)(MeOH)^I^		1.709	2.248	2.337	155.24	105.93
		1.709			96.81	
[MoO_2_(sae)(MeOH)]^a^		1.697	2.252	2.339	158.9	106.1
		1.704			94.2	
[MoO_2_(cysS-OR)(MeOH)]^b^		1.699	2.291	2.385	160.8	105.9
		1.709			92.5	
[MoO_2_(hbhy)(EtOH)]^c^		1.696	2.234	2.356	158.44	106.03
		1.699			93.84	
[MoO_2_(doin)(MeOH)]^d^		1.700	2.250	2.331	159.54	106.65
		1.714			93.16	
[MoO_2_(hpmp)(MeOH)]^e^		1.703	2.274	2.355	159.38	107.05
		1.709			93.07	
[MoO_2_(hpemp)(MeOH)]^f^		1.707	2.268	2.387	159.81	106.36
		1.699			93.53	
[MoO_2_(moip)(MeOH)]^g^		1.700	2.251	2.349	158.82	105.61
		1.702			95.01	
[MoO_2_(ssh)(MeOH)]^h^		1.692	2.234	2.349	157.6	106.4
		1.705			93.7	
[MoO_2_(hmt)(MeOH)]^i^		1.727	2.273	2.351	156.41	105.26
		1.704			95.29	
MoO_2_(bhpd)(MeOH)^j^		1.988	2.284	2.301	160.49	105.9
		1.717			90.93	

Notes: (1) solvent = MeOH or EtOH. References: (I) this work; (a) Glowiak *et al.* (2003); (b) Liimatainen *et al.* (2000); (c) Dinda *et al.*(2006); (d) Niaz *et al.* (2010); (e) Sheikhshoaie *et al.* (2009); (f) Rezaeifard *et al.* (2010); (g) Saeednia *et al.* (2009); (h) Rao *et al.* (1999); (i) Vrdoljak *et al.* (2010); (j) Pramaniky *et al.* (2007).

### Table 4 Comparison of important absorption band frequencies (cm^-1^) in some related dioxidomolybdenium(VI) complexes

**Table:**

Complex		ν_s_(Mo═O)^1^	ν_s_(Mo═O)^2^	ν(C═N)
[MoO_2_(bnms)(MeOH)^I^		939	915	1627
[MoO_2_(sae)(MeOH)]^a^		928	906	1641
[MoO_2_(cysS-OR)(MeOH)]^b^		995	980	1626
[MoO_2_(hbhy)(EtOH)]^c^		-	-	-
[MoO_2_(doin)(MeOH)]^d^		-	-	-
[MoO_2_(hpmp)(MeOH)]^e^		924	900	1638
[MoO_2_(hpemp)(MeOH)]^f^		920	909	1638
[MoO_2_(moip)(MeOH)]^g^		-	-	-
[MoO_2_(ssh)(MeOH)]^h^		939	911	1637
[MoO_2_(hmt)(MeOH)]^i^		932	901	1626
MoO_2_(bhpd)(MeOH)^j^		939	914	1579

Notes: (1) ν symmetry; (2) ν asymmetry References: (I) this work; (a) Glowiak *et al.* (2003); (b) Liimatainen *et al.* (2000); (c) Dinda *et al.*(2006); (d) Niaz *et al.* (2010); (e) Sheikhshoaie *et al.* (2009); (f) Rezaeifard *et al.* (2010); (g) Saeednia *et al.* (2009); (h) Rao *et al.* (1999); (i) Vrdoljak *et al.* (2010); (j) Pramaniky *et al.* (2007).
